# Modulation of nociceptive nerve activity as a novel therapeutic strategy in acute myeloid leukemia

**DOI:** 10.1016/j.fmre.2026.03.012

**Published:** 2026-03-28

**Authors:** Honglin Duan, Pujiao Li, Linmin Zhang, Yanling Lv, Meng Yang, Yicheng He, Shangda Yang, Shihong Lu, Tao Cheng, Hui Cheng

**Affiliations:** a State Key Laboratory of Experimental Hematology, Haihe Laboratory of Cell Ecosystem, National Clinical Research Center for Blood Diseases, Institute of Hematology & Blood Diseases Hospital, Chinese Academy of Medical Sciences & Peking Union Medical College, Tianjin 300020, China; b Center for Stem Cell Medicine, Department of Stem Cell & Regenerative Medicine, Chinese Academy of Medical Sciences, Tianjin 300020, China

**Keywords:** Nociceptive nerve, Acute myeloid leukemia, Bone marrow microenvironment, Neural remodeling, Dietary capsaicin

## Abstract

Emerging evidence has highlighted a critical role of the neuro-microenvironment in regulating hematopoiesis and hematologic malignancies. However, how malignant cells remodel the neural compartment and how these neural changes in turn influence disease progression remain poorly understood. In this study, we mapped the spatial distribution of sympathetic (TH^+^) and nociceptive (CGRP^+^) nerve fibers in the bone marrow and spleen under steady-state conditions and during acute myeloid leukemia (AML) using immunofluorescence imaging. We found that AML induces a reduction of nerve fiber density at the early stage of AML and that leukemic cells preferentially accumulate in neural-sparse regions. Functional investigations revealed that pharmacological ablation of nociceptive nerves via capsaicin accelerated leukemic progression and disrupted stressed hematopoiesis. Conversely, activation of nociceptive nerves by dietary capsaicin delayed AML onset and improved overall survival. Further mechanistic investigation revealed that the CGRP–RAMP1 axis inhibits leukemia progression by suppressing the TNF-α pathway in AML cells. These findings highlight the anti-leukemic role of nociceptive nerves and suggest therapeutic potential for neural modulation in AML.

## Highlights

• AML remodels the neural microenvironment early in disease progression.
• Leukemic cells preferentially accumulate in neural-sparse regions in bone marrow.
• Pharmacological ablation of nociceptive nerves accelerates leukemic progression and disrupts stressed hematopoiesis.
• Activation of nociceptive nerves by dietary capsaicin delayed AML onset and improved overall survival.

## Graphical abstract

**Figure:**
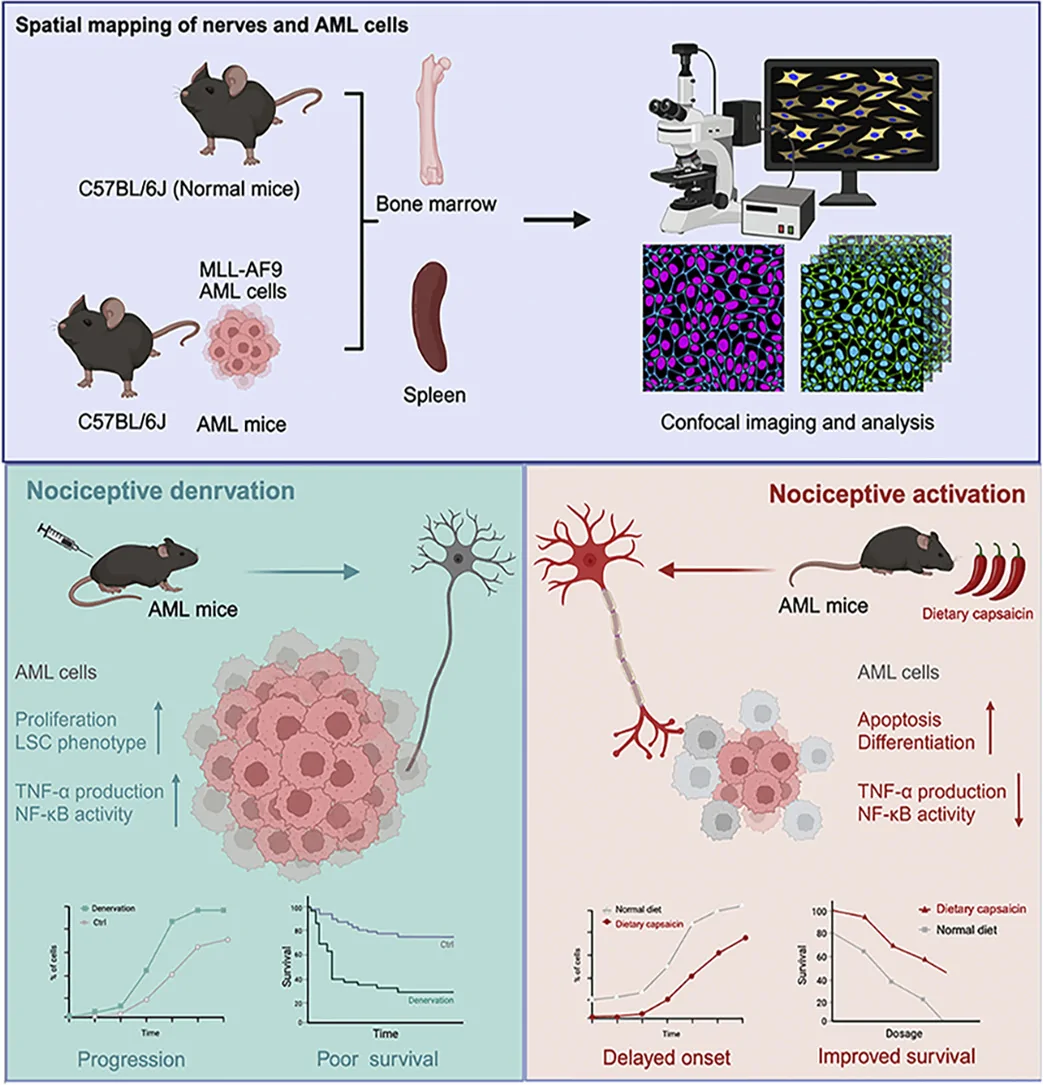

## 1 Introduction

The neuro-microenvironment represents an integral component of the hematopoietic niche within bone marrow (BM) and spleen [1,2]. It is primarily composed of sympathetic and nociceptive nerves, playing distinct yet coordinated roles in regulating hematopoiesis [3,4]. Sympathetic nerves are known to modulate circadian hematopoietic rhythms through neurotransmitter release and facilitate hematopoietic stem/progenitor cells (HSPCs) mobilization in concert with granulocyte colony-stimulating factor (G-CSF) directly on HSPCs or indirectly via stromal cells [[5], [6], [7], [8], [9]]. In parallel, nociceptive nerves, which release calcitonin gene-related peptide (CGRP), contribute to HSPC mobilization through CGRP-mediated activation of the calcitonin receptor-like receptor (CALCRL) and intracellular RAMP1 signaling [10,11].

Acute myeloid leukemia (AML) is the most common and aggressive hematologic malignancy. Leukemia progression involves profound remodeling of the BM and splenic microenvironments, which not only disrupts normal hematopoiesis but may also actively support leukemic progression by generating a permissive niche for malignant cells [12,13]. Growing evidence implicates sympathetic nerve dysfunction in AML development [8]. Likewise, nociceptive nerves have been shown to modulate tumor pathogenesis in solid malignancies such as melanoma and oral squamous cell carcinoma [14,15]. Additionally, CALCRL signaling has been linked to drug resistance and relapse in leukemia [16,17]. Despite these advances, the specific role of nociceptive nerves in AML remains poorly understood.

To address this gap, we employed an MLL-AF9-driven AML mouse model to investigate how leukemic cells remodel the neuro-microenvironment in BM and spleen, and how such neural changes in turn influence leukemic progression. Using immunofluorescence imaging, we systematically characterized the spatial distribution and density of neural fibers in healthy and leukemic tissues at distinct disease stages. Furthermore, we modulated nociceptive nerve activity through either high-dose capsaicin-mediated ablation or dietary capsaicin to evaluate its functional impact on both leukemic and normal hematopoietic cells. Through assessments of leukemic burden, survival, HSPC dynamics, and cellular apoptosis/proliferation, this study aims to elucidate the role of nociceptive nerves in AML progression and explore their therapeutic potential.

## 2 Material and methods

### 2.1 Mice

Adult 8 to 10-week-old C57BL/6-Ly5.2 (Ly5.2, CD45.2) mice of both genders were purchased from Beijing Vital River Laboratory Animal Technology. The *Tcf21* CreER; TdTomato mice and *lepr* Cre; TdTomato mice involved in this study were kindly provided by Professor Bo Zhou from the Institute of Biochemistry and Cell Biology, Chinese Academy of Sciences. Cas9-eGFP knock-in mice were purchased from the Jackson Laboratory. All animal experiments were approved by the Animal Ethical and Welfare Committee of the Institute of Hematology, Chinese Academy of Medical Sciences (Approval No. IHCAMS-DWLL-NSFC2024154-1). All mice were housed in specific pathogen-free facilities.

### 2.2 Construction of MLL-AF9 induced mouse models

To establish a murine model of MLL-AF9 leukemia that allows for reliable distinction between donor leukemia cells and recipient-derived hematopoietic cells, four distinct types of donor cells were used: c-kit^+^ cells isolated from CD45.1^+^ mice, Actin-GFP transgenic mice, Cas9-eGFP knock-in mice, and Fucci mice. Retrovirus was produced by transfecting 293T cells with MLL-AF9, pKat, and pVSV-G plasmids using Lipofectamine 2000. Supernatants were collected at 48 and 72 h, concentrated via Amicon filtration, and used to transduce c-kit^+^ cells. Forty-eight hours post-transduction, transduced cells were transplanted into lethally irradiated recipients via tail vein injection. Once the peripheral blast count exceeded 40%, primary (P1) leukemia cells were harvested from bone marrow and spleen and transplanted into sublethally irradiated secondary recipients. Secondary (P2) leukemic cells were collected upon disease progression and used for subsequent transplants (1 × 10^5^ cells/mouse, i.v.).

### 2.3 Construction of RAMP1-KO MLL-AF9 cells

c-kit⁺ cells isolated from Cas9-eGFP knock-in mice were harvested and transduced to establish the MLL-AF9 leukemia model. These Cas9-eGFP⁺ AML cells were subsequently infected with lentiviral sgRNA-mCherry constructs targeting *Ramp1*. The sgRNAs used in this study were: sgRNA-1: CACCGCTGGAGGAGCAAGCGCACAG; sgRNA-2: CACCGACTTGCTGAAGTATCGATGG.

### 2.4 Homing assay of AML cells

To assess the homing capacity of leukemia cells to the BM, a homing assay was performed as follows. First, GFP-labeled MLL-AF9 leukemia cells (1 × 10^5^ cells per mouse) were transplanted into recipient mice via tail vein injection. The mice were then sacrificed 16 h post-transplantation. BM cells were harvested from both femurs and tibias by flushing with cold PBS buffer containing 2% fetal bovine serum (PBE). The percentage of GFP⁺ leukemia cells in the BM was quantified using flow cytometry. The absolute number of homed leukemia cells was calculated based on the total cell count and the percentage of GFP⁺ cells.

### 2.5 Tissue processing and immunostaining

Mouse femurs and spleens were fixed in 4% PFA overnight at 4 °C, dehydrated in 15% and 30% sucrose (24 h each), embedded in OCT, and stored at −80 °C. For arterial labeling, anti-CD144-AF647 (7.5 μg) was injected intravenously 10 min prior to sacrifice. Cryosections (20 μm) were prepared using the Kawamoto film method. Sections and whole-mount half-bones were blocked in 20% donkey serum with 0.5% Triton X-100/PBS (2 h, RT or overnight at 4 °C), then incubated for 72 h at 4 °C with primary antibodies (anti-TH, anti-CGRP, anti-TUBB3-AF488) in 10% donkey serum with 0.1% Triton X-100/PBS. After extensive PBS washes, samples were stained with DAPI and secondary antibodies (2 h, RT).

### 2.6 In vivo treatments

For nociceptive denervation experiments, mice received three escalating doses of capsaicin (30 mg/kg, 70 mg/kg and 100 mg/kg body weight) or vehicles for 3 consecutive days. Capsaicin (HY-10,448, MCE) was dissolved in 10% ethanol, 10% Tween-80 and 80% sterile water and subsequently injected into the subcutaneous fat tissues of the back after anesthesia in mice. To activate nociceptive nerves, mice were fed with dietary capsaicin (food containing capsaicin, 100 mg/kg) 1 week before transplantation and throughout the entire course of AML. For tamoxifen induction, *Tcf21* CreER; TdTomato mice (typically 5 weeks old) were administered tamoxifen (T5648, Sigma-Aldrich) intraperitoneally (i.p.) at 100 mg/kg for 5 consecutive days, and spleen samples were collected 4 weeks after tamoxifen induction.

### 2.7 Cell isolation and flow cytometry

BM cells were flushed from the femurs using ice-cold PBE buffer. Spleens were mechanically dissociated and passed through a 70-μm nylon mesh to obtain single-cell suspensions. Cells were stained with a cocktail of fluorescently conjugated antibodies. After incubation for 30 min at 4 °C, cells were washed with PBE and resuspended in 300–500 μL of buffer. Flow cytometry was performed using BD FACS Aria or Agilent NovoCyte instruments. Data were analyzed with FlowJo (v10.4.0) and NovoExpress software.

### 2.8 Cell-cycle analyses and Annexin V staining

For cell-cycle analyses of AML cells, MA9-GFP cells from BM and SP were sorted and processed according to the manufacturer’s instructions (BD IntraSure™ Kit), subsequently stained with mKi-67 (Biolegend) for 30 min at room temperature and Hoechst 33,342 (Molecular Probes) at 1 μg/mL before detection. For apoptosis analyses of AML cells and LSCs, cells from BM and SP were processed according to the manufacturer’s instructions (Annexin V-PE Apoptosis detection kit, BD Biosciences). In vivo, Fucci AML cells were introduced to indicate the cell cycle of AML cells.

### 2.9 ELISA

Two femurs were flushed with 500 μL PBS containing protease inhibitor cocktail (cOmplete ULTRA, Merck). The suspension was then centrifuged. The supernatant was harvested for ELISA analysis immediately using the CGRP EIA Kit (Cayman Chemical) or the TNF-α ELISA Kit (Invitrogen) according to the manufacturer’s instructions.

### 2.10 RNA-seq processing and analysis

A total of 10,000–20,000 MA9-GFP⁺ AML cells were sorted by FACS. Total RNA was extracted using TRIzol reagent. mRNA libraries were prepared with the TruePrep DNA Library Prep Kit V2 for Illumina and sequenced on an Illumina NovaSeq 6000 platform. Raw reads were quality-checked with FastQC (v0.11.9), and adapter, poly-N, and low-quality sequences were filtered to obtain clean reads. Differential gene expression analysis was performed using a threshold of |log_2_FC| ≥ 1 and FDR < 0.05. Functional enrichment for GO biological processes and KEGG pathways was conducted with FDR and *P*-value cutoffs of < 0.05.

### 2.11 Western blotting assays

Briefly, cellular extracts were harvested from an equal number and resuspended in 5× SDS-PAGE loading buffer. Protein samples were boiled, separated by SDS-PAGE, and transferred to a PVDF membrane (Merck Millipore), followed by immunoblotting with primary and secondary antibodies.

### 2.12 Statistical analysis

The GraphPad Prism 10.0 software was used for statistical analysis. Unpaired t tests and analysis of variance were used to generate *P* values for most of the datasets. Differences at *P* < 0.05 were considered significant.

## 3 Results

### 3.1 Spatial mapping of neural architecture in bone marrow reveals region-specific innervation

To characterize neural distribution in healthy murine BM, we performed immunofluorescence imaging on cryosections and whole-mount half-bones using pan-neuronal (Tubb3), sympathetic (TH), and nociceptive (CGRP) markers. Our analysis revealed extensive co-localization of TH⁺ and CGRP⁺ fibers with Tubb3⁺ structures, as well as substantial overlap between sympathetic and nociceptive fibers, indicating coordinated neural patterning in BM (Fig. 1A). Regional analysis based on anatomical landmarks showed the highest nerve density in the diaphysis, followed by the proximal metaphysis, proximal epiphysis, and femoral head, while distal epiphysis and distal metaphysis were largely devoid of neural innervation, suggesting a regionally specialized microenvironment (Fig. 1B–D). Furthermore, nerve fibers are closely aligned with arterial vessels, suggesting potential neuro-vascular interactions in hematopoietic regulation (Fig. 1E). Together, these data indicate that sympathetic and nociceptive nerves are mainly distributed in the proximal femur but not the distal metaphysis region, along with arteries in the BM.

**Fig. 1:**
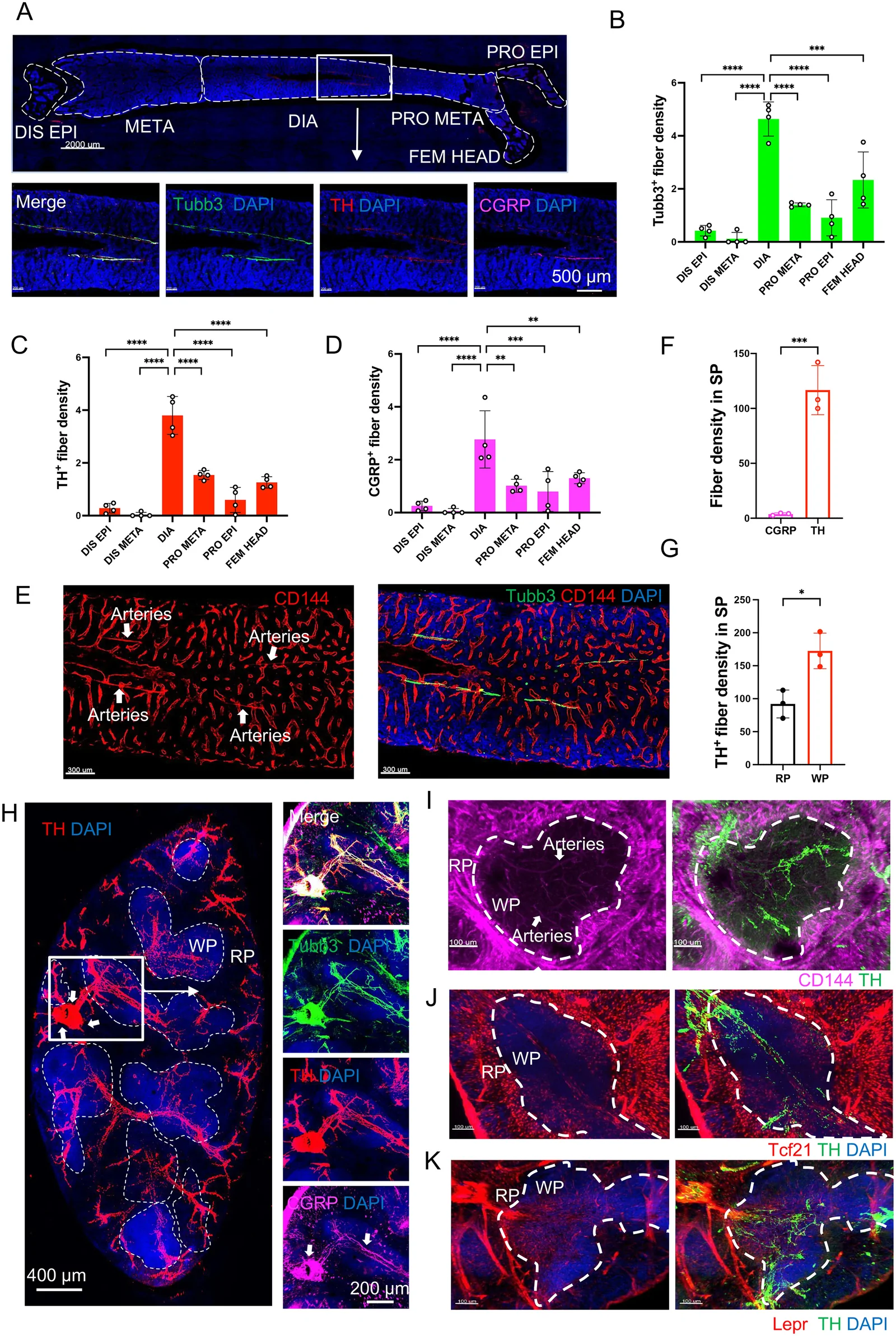
**Mapping the spatial distribution of nerve fibers in BM and SP under homeostasis.** (A) The BM is divided into different parts, including proximal epiphysis (PRO EPI), proximal metaphysis (PRO META), diaphysis (DIA), distal metaphysis (META), distal epiphysis (DIS EPI) and femoral head (FEM HEAD). (B-D) Femoral BM innervation quantified by Tubb3^+^ (B), TH^+^ (C) and CGRP^+^ (D) stained nerve fiber area divided by BM area (*n* = 4). (E) Representative confocal images of nerve fibers and arteries in the BM. (F) Quantification of TH^+^ and CGRP^+^ nerve fiber density in SP (*n* = 3). (G) Quantification of TH^+^nerve fiber density in white pulp (WP) and red pulp (RP) of SP (*n* = 3). (H) Representative confocal images of nerve fiber in SP. (I-K) Representative confocal images of colocalization of TH^+^ nerve fiber with CD144^+^ endothelial cells (I), *Tcf21*^+^ stromal cells (J), *Lepr*^+^ stromal cells (K) in SP. Statistical analysis: (B–D) 2-way ANOVA multiple; (F, G) unpaired t-test. Data are presented as mean ± SD. Significance: *P < 0.05, **P < 0.01, ***P < 0.001, ****P < 0.0001.

### 3.2 Splenic neural landscape is dominated by sympathetic innervation and exhibits compartmentalization

To characterize the neural architecture in the spleen of healthy mice, we prepared 400 μm-thick tissue sections using a vibratome, followed by immunofluorescence imaging. As a visceral organ, the spleen exhibited a low density of CGRP⁺ nerve fibers, with CGRP⁺ signal detected in major nerve tracts. In contrast, abundant TH⁺ nerve fibers were observed. Quantitative analysis revealed that TH⁺ nerve fiber density was significantly higher than CGRP⁺ fibers (Fig. 1F). Anatomically, the spleen is divided into white pulp (WP) and red pulp (RP). Both compartments contained TH⁺ nerve fibers, and further quantification showed that the TH⁺ fiber density was significantly higher in WP than RP (Fig. 1G–H). Given the established roles of endothelial cells, *Tcf21*⁺ stromal cells, and *Lepr*⁺ stromal cells as key regulators of hematopoiesis [[18], [19], [20], [21]], we investigated their spatial relationships with neural components (Fig. 1I–K). Staining of endothelial cells and nerve fibers revealed that TH⁺ nerve fibers in the WP are closely surrounded by arterial endothelial cells (Fig. 1I). Using *Tcf21* CreER; TdTomato reporter mice, we observed that *Tcf21*⁺ cells in the WP exhibited clear co-localization with TH⁺ nerve fibers (Fig. 1J). In addition, using *Lepr* Cre; TdTomato reporter mice, we observed *Lepr*⁺ cells were mainly located in the WP and frequently appeared intertwined with TH⁺ nerve fibers (Fig. 1K). These results indicate nerve fibers exhibit extensive spatial interaction with other niche cells in SP, suggesting their collaborative role in the regulation of hematopoiesis.

### 3.3 AML remodels the neuro-microenvironment at the early stage of AML

To investigate neural remodeling in AML, we generated an MLL-AF9-driven leukemia model and classified disease progression by peripheral AML percentage. Femoral bone samples at different stages of leukemia progression (2%, 5%, 20%, 40%, 60%, and 80%) were collected, followed by immunofluorescence staining of nerve fibers (Fig. 2A). Immunofluorescence and quantitative analysis revealed a significant reduction in Tubb3⁺, TH⁺, and CGRP⁺ nerve densities as early as the 2% AML stage (Fig. 2B–D). TH⁺ fibers were more profoundly affected than CGRP⁺ fibers in early disease stages, with TH⁺ innervation nearly absent in BM (Fig. 2A–D). Notably, although CGRP⁺ fiber density was also significantly reduced, these fibers persisted in BM until the end-stage of leukemia (AML 80%) (Fig. 2D), suggesting sustained nociceptive signaling throughout leukemic progression. Parallel analysis of splenic sections showed a similar dramatic loss of both TH⁺ and CGRP⁺ innervation early in leukemia (Fig. 2E–G). These results demonstrate that remodeling of the neuro-microenvironment occurs during early leukemic stages, characterized by a pronounced reduction of nerve density.

**Fig. 2:**
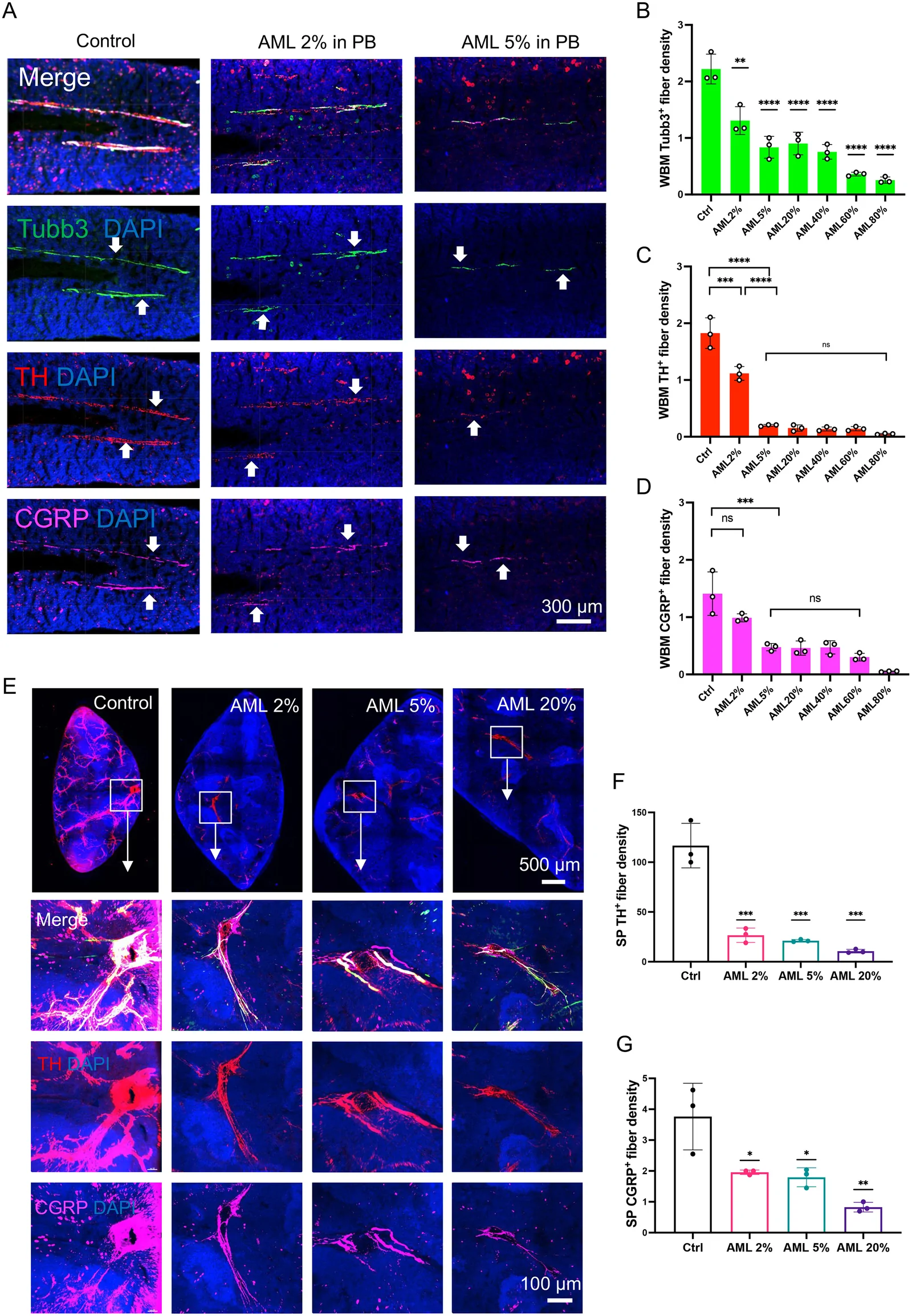
**Neuro-microenvironment remodeling under AML.** (A) Representative confocal images of Tubb3^+^, TH^+^ and CGRP^+^nerve fibers under AML. (B-D) Femoral BM innervation quantified by Tubb3^+^ (B), TH^+^ (C) and CGRP^+^ (D) stained nerve fiber area divided by BM area at different stages of AML (*n* = 3). (E) Representative confocal images of nerve fiber in SP at different stages of AML. (F-G) Quantification of TH^+^ (F) and CGRP^+^ (G) nerve fiber density in SP at different stages of AML (*n* = 3). Statistical analysis: 2-way ANOVA multiple. Data are presented as mean ± SD. Significance: ns = not significant, *P < 0.05, **P < 0.01, ***P < 0.001, ****P < 0.0001.

### 3.4 AML cells accumulate preferentially in neural-sparse regions

Interestingly, in alignment with the spatial distribution of nerves in BM, we also identified a distinct spatial patterning of AML cells. During early leukemic progression (AML 5%), the distal metaphysis of the femurs exhibited a pale appearance compared to the reddish hue of the proximal diaphysis (Fig. 3A). Flow cytometric analysis of mechanically separated femoral regions confirmed significantly higher leukemic burden in the metaphysis than in the diaphysis (72.8 ± 2.0% vs. 41.8 ± 5.8%, respectively) (Fig. 3B). To further assess proliferative activity within these distinct niches, we employed a Fucci reporter system, enabling visualization of cell-cycle status and spatial distribution: cells in G₀/G₁ phase (quiescent) emit red fluorescence, while those in S/G₂/M phase (proliferating) emit green fluorescence. Using both flow cytometry and confocal imaging, we detected a higher percentage of proliferating AML cells in the metaphysis compared to the diaphysis (Fig. 3C–E). These findings demonstrate that the metaphysis—a region largely devoid of neural input—constitutes a favorable niche that supports the expansion of AML cells during early disease progression.

**Fig. 3:**
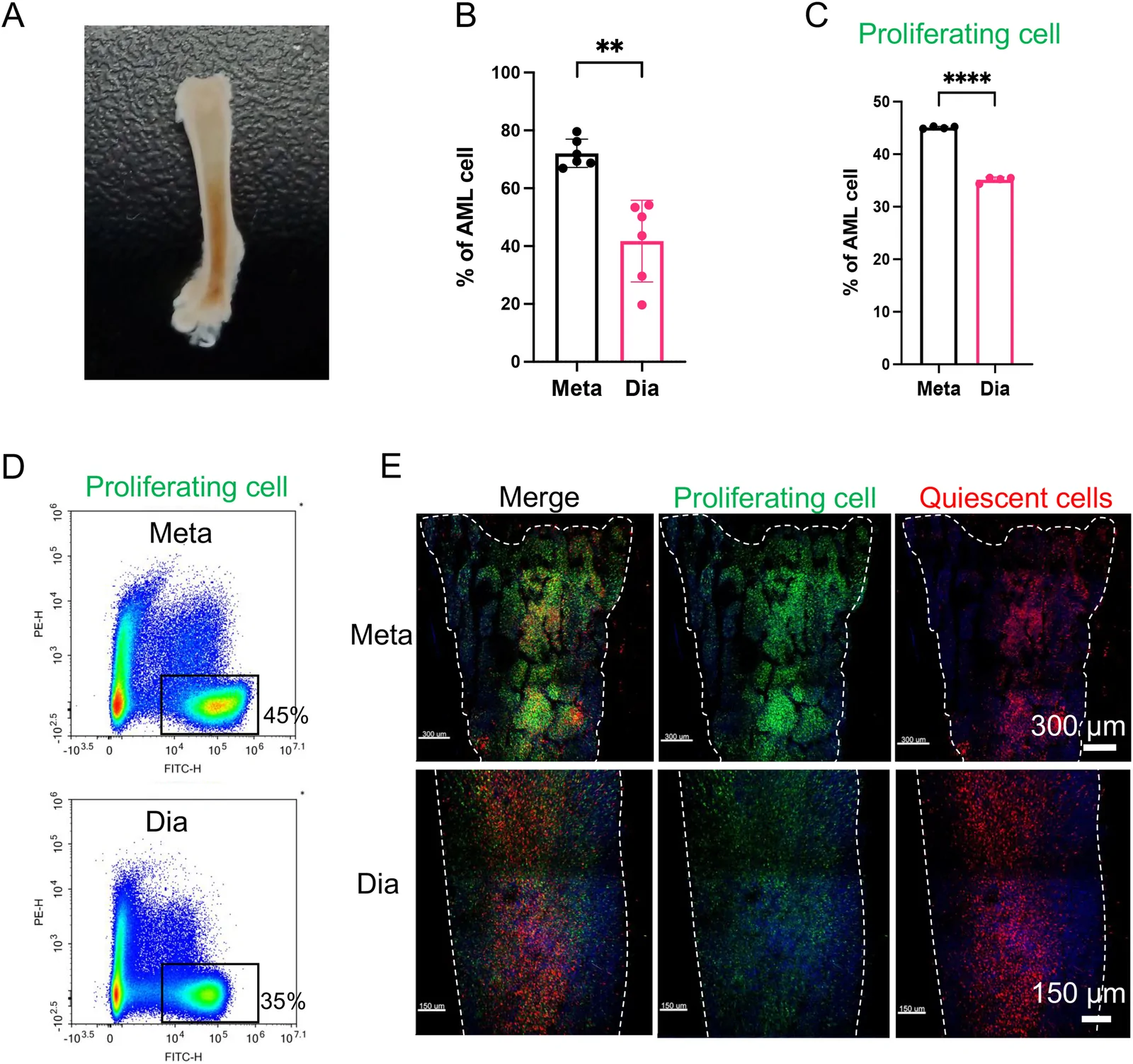
**Variation of AML cells’ proliferation in distinct neural niches.** (A) Representative images of bone at the early stage of AML. (B) Frequency of AML cells in metaphysis (Meta) and diaphysis (Dia) at the early stage of AML (*n* = 6). (C) Frequency of proliferative AML cells in metaphysis (Meta) and diaphysis (Dia) at the early stage of AML (*n* = 4). (D) Representative flow cytometry plots showing the frequency of proliferative AML cells in metaphysis (Meta) and diaphysis (Dia) at the early stage of AML. (E) Representative confocal images showing the proliferating (green) and quiescent (red) AML cells in Meta and Dia at the early stage of AML. Statistical analysis: unpaired t-test. Data are presented as mean ± SD. Significance: *P < 0.05, **P < 0.01, ***P < 0.001, ****P < 0.0001.

### 3.5 Sympathetic denervation promotes leukemic infiltration in SP but not BM during early AML

To investigate the impact of sympathetic nerve remodeling on AML progression, we chemically ablated sympathetic signaling using 6-hydroxydopamine (6-OHDA) to model leukemia-associated sympathetic neuropathy (Fig. S1A). Sympathetic denervation accelerated leukemia progression, as evidenced by increased leukemic burden in PB and significantly reduced overall survival (Fig. S1B–C). Since AML-mediated sympathetic remodeling primarily occurs during early disease stages as previously described, we focused on early time points to evaluate leukemic infiltration in BM and SP and quantify leukemia stem cell (LSC), defined as IL-7R^−^Lin^−^GFP^+^c-kit^hi^CD34^lo^CD16/32^hi^ granulocyte-macrophage progenitors (L-GMPs). Notably, while no significant differences were observed in either AML cell proportion or LSC counts within BM (Fig. S1D–E), the spleens of 6-OHDA-treated mice exhibited significant enlargement along with elevated AML cell percentages and LSC numbers compared to controls (Fig. S1F–I). These findings indicate that sympathetic denervation enhances leukemic infiltration specifically in the spleen, but not in the BM, during the early phase of AML development.

### 3.6 Nociceptive denervation facilitates leukemic invasion in BM and SP

We next sought to investigate the function of nociceptive nerves in AML progression. To mimic leukemia-associated neuropathy, we pharmacologically ablated these nerves using high-dose subcutaneous capsaicin injections (CAP SC.) prior to leukemia induction (Fig. 4A). Immunofluorescence confirmed selective depletion of nociceptive fibers, with Tubb3⁺ and TH⁺ nerves remaining intact (Fig. 4B). Homing assays revealed no significant difference in initial AML cell recruitment between groups (Fig. 4C), indicating that differential homing did not account for subsequent phenotypic differences. Our results demonstrated that nociceptive denervation accelerated leukemic progression and shortened overall survival (Fig. 4D–E). On day 17 post-transplantation, CAP SC. mice showed significantly increased AML burden in both BM (26.27 ± 1.67% vs. 10.88 ± 0.98%) and SP (23.40 ± 1.96% vs. 11.21 ± 0.83%), alongside splenomegaly (Fig. 4F–H; S2A–B).

**Fig. 4:**
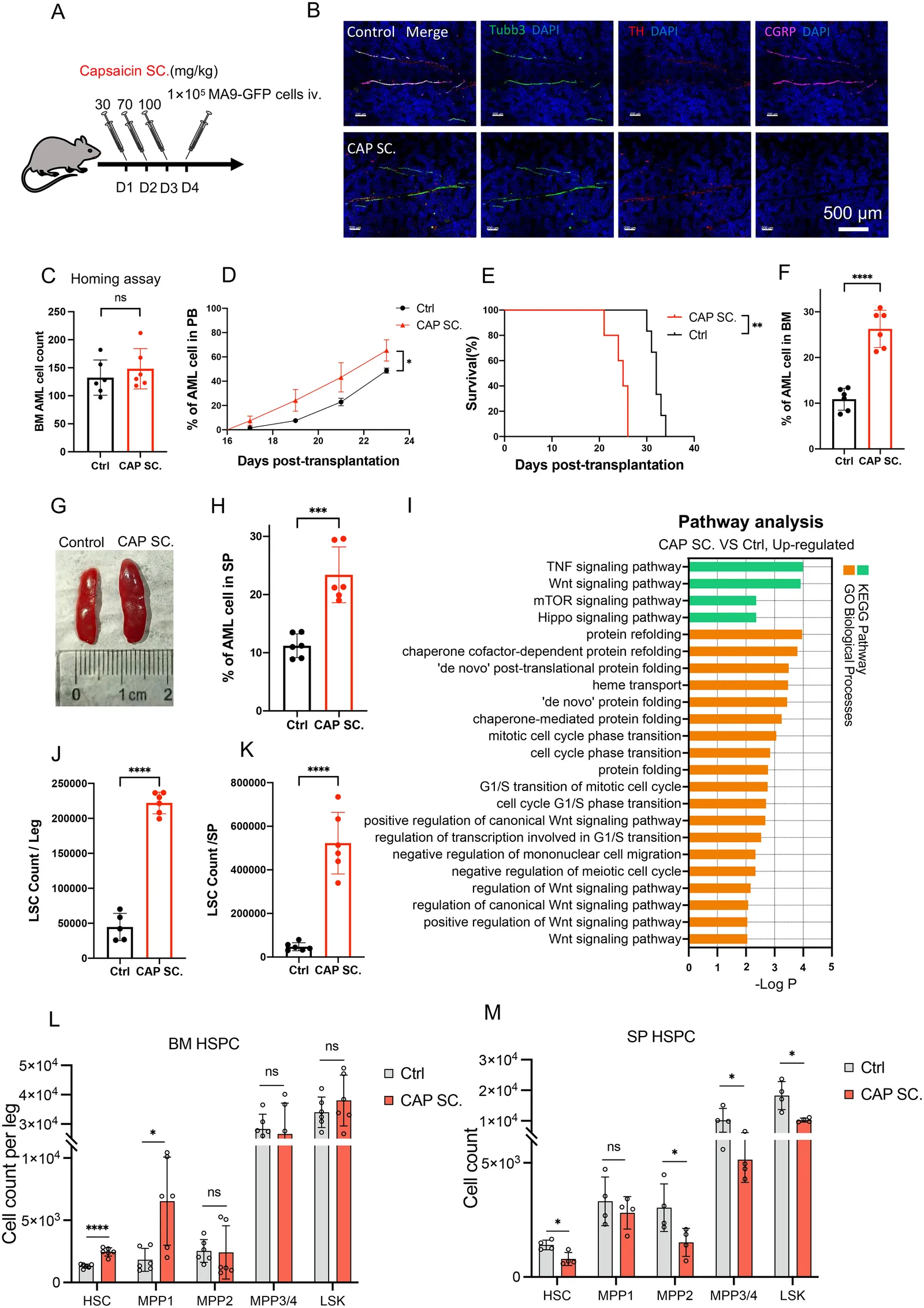
**Nociceptive denervation facilitates invasion of AML cells and disturbs stressed hematopoiesis.** (A) Schematic of denervation of nociceptive nerves by capsaicin. (B) Representative confocal images showing Tubb3^+^, TH^+^ and CGRP^+^ nerve fibers in the CAP SC. group and the control group. (C) BM AML cell count in the homing assay (*n* = 6). (D) Onset of AML monitored by detecting the frequency of AML cells in PB (*n* = 4 Ctrl mice, *n* = 6 CAP SC. mice). (E) Overall survival of control and capsaicin SC. treated AML models (*n* = 6 Ctrl mice, *n* = 5 CAP SC. mice). (F) Frequency of AML cells in the BM 17 days post-transplantation (*n* = 6). (G) Representative image of the spleen 17 days post-transplantation. (H) Frequency of AML cells in the SP 17 days post-transplantation (*n* = 6). (I) KEGG and GO pathway analysis of upregulated DEGs in CAP SC. versus control. (J-K) LSC count per leg (J) or SP (K) 17 days post-transplantation (*n* = 6). (L-M) HSPC count of BM (L) and SP (M) 17 days post-transplantation (*n* = 6 for BM, *n* = 4 for SP). Statistical analysis: (C, F, H, J, K, L, M) unpaired t-test; (D) paired t-test; (E) survival curve comparison. Data are presented as mean ± SD. Significance: *P < 0.05, **P < 0.01, ***P < 0.001, ****P < 0.0001.

To further elucidate alterations of AML cells following nociceptive nerve ablation, we performed RNA-seq on BM AML cells. Compared to the control, the CAP SC. group exhibited upregulation of 454 genes and downregulation of 1345 genes (Fig. S2C). Upregulated pathways included tumor necrosis factor (TNF) and Wnt signaling, as well as mitotic cell cycle transition, supporting enhanced proliferation and invasion of AML cells after nociceptive denervation (Fig. 4I). Conversely, downregulated pathways were primarily related to the production of immune mediators, positive regulation of immune response, and cytokine production (Fig. S2D), indicating suppression of immunity in the absence of nociceptive signaling. Furthermore, pathways involved in hematopoietic cell lineage and leukocyte differentiation were suppressed in the CAP SC. group (Fig. S2D), which indicated impaired differentiation of LSC. We further validated the elevated LSC burden after denervation (Fig. S3A). LSC counts were markedly elevated in both the BM (4.97-fold) and spleen (11.05-fold) of CAP SC. mice (Fig. 4J–K; Fig. S3A). Collectively, these results demonstrate that pharmacological ablation of nociceptive nerves promotes leukemic expansion by enhancing proliferation and suppressing immune surveillance.

### 3.7 Nociceptive denervation disturbs stressed hematopoiesis under AML

To examine the role of nociceptive nerves in regulating hematopoiesis during leukemia, we quantified multiple HSPC populations within the MA9-GFP⁻ compartment of leukemic mice using flow cytometry (Fig. S3B–C). The number of HSCs per leg was significantly higher in CAP SC. mice (2456 ± 136) compared to controls (1316 ± 68), representing a 1.87-fold increase, despite the elevated leukemic burden in the BM of CAP SC. mice (Fig. 4L). Similarly, MPP1 cells were markedly increased in the CAP SC. group (6538 ± 1447) relative to controls (1821 ± 377), corresponding to a 3.59-fold difference in BM (Fig. 4L). In contrast, splenic HSCs were reduced in CAP SC. mice (788 ± 141) compared to controls (1404 ± 106). Counts of MPP1, MPP2, and MPP3/4 were also consistently lower in the spleens of CAP SC. mice (Fig. 4M). These findings indicate that nociceptive denervation resulted in pronounced retention of HSCs and MPP1 cells within BM, coupled with a reduction in splenic HSPCs. Together, these data suggest that impaired mobilization of hematopoietic cells occurs in the absence of nociceptive signaling, ultimately suppressing extramedullary hematopoiesis under leukemic stress.

### 3.8 Dietary capsaicin delays the onset of AML and improves the overall survival

Having identified the potential anti-leukemia effect of nociceptive nerves, we further investigated the feasibility and efficacy of targeting these fibers. Whereas high-dose subcutaneous capsaicin inactivates nociceptive nerves, low-dose capsaicin can activate TRPV1 on these fibers and enhance their function [10,22,23]. To explore the effect of nociceptive nerve activation on AML progression, we implemented a dietary capsaicin intervention (CAP diet) (Fig. 5A). Consistent with previous studies, dietary capsaicin administration did not significantly affect the body weight of the mice (Fig. S4A). ELISA confirmed elevated CGRP release in BM, and immunofluorescence analysis revealed higher CGRP MFI, together confirming the successful activation of nociceptive nerves. (Fig. 5B; Fig. S4B). Homing assays showed no significant difference between groups (Fig. 5C), ruling out homing effects as a contributor to differential leukemic progression. Our results showed that disease progression was delayed and overall survival was extended in capsaicin-fed mice (Fig. 5D–E). On day 18, the proportion of AML cells in the BM was significantly lower in the CAP diet group (18.57 ± 0.69%) than in controls (39.80 ± 0.80%) (Fig. 5F). Spleens from capsaicin-fed mice were also noticeably smaller (Fig. 5G), with lower percentages of AML cells than controls (Fig. 5H). These results demonstrate that activation of nociceptive nerves through dietary capsaicin effectively attenuates leukemic burden in both the BM and spleen.

**Fig. 5:**
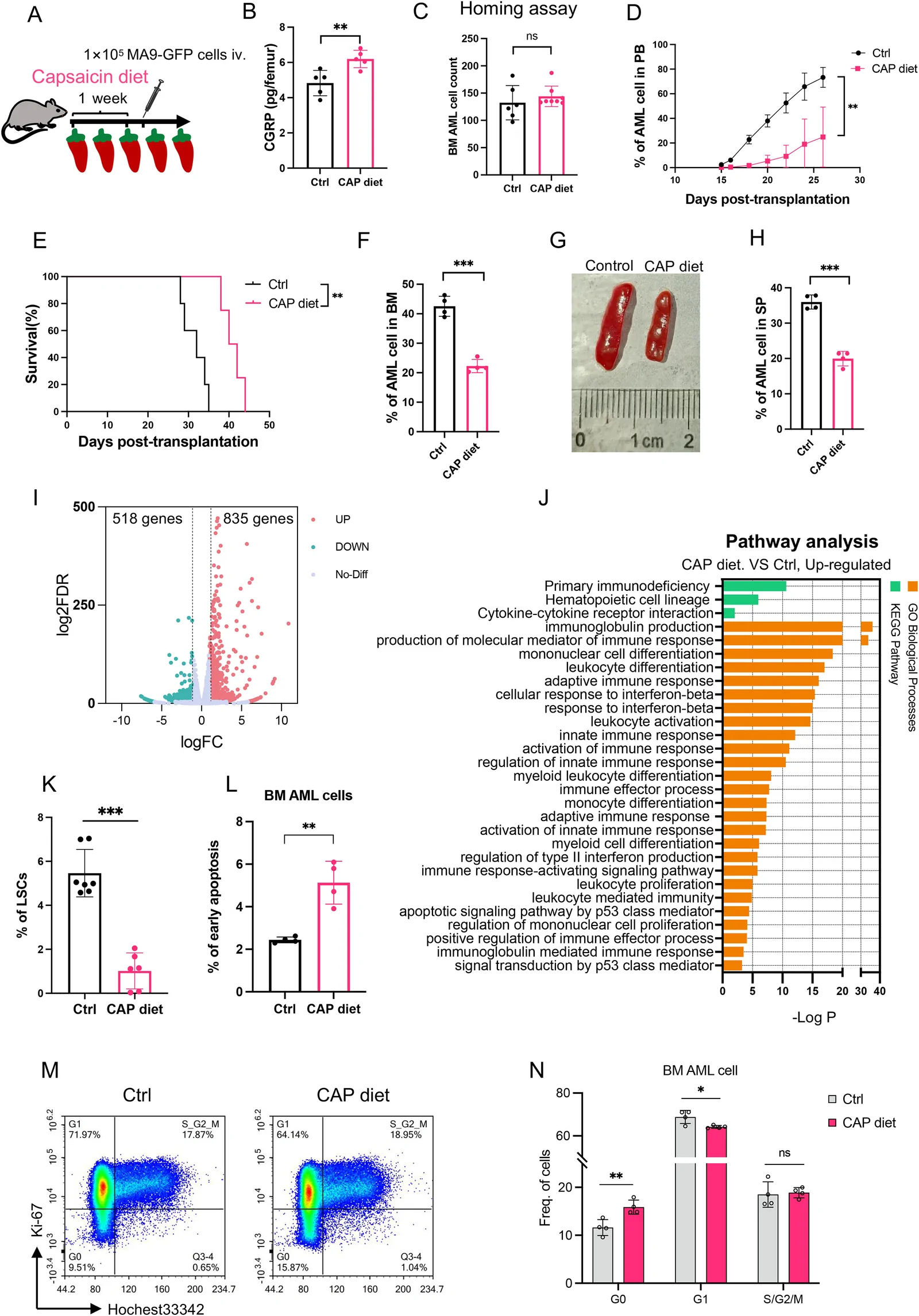
**Activation of nociceptive nerves by dietary capsaicin attenuates leukemic progression.** (A) Schematic of activation of nociceptive nerves by capsaicin diet. (B) Concentration of CGRP in BMEF (*n* = 5). (C) BM AML cell count in the homing assay (*n* = 6 Ctrl mice, *n* = 8 CAP diet mice). (D) Onset of AML monitored by detecting the frequency of AML cells in PB (*n* = 4–5). (E) Overall survival of control and capsaicin diet AML models (*n* = 5). (F) Frequency of AML cells in BM 18 days post-transplantation (*n* = 4). (G) Representative image of spleen 18 days post-transplantation. (H) Frequency of AML cells in SP 18 days post-transplantation (*n* = 4). (I) Volcano plots showing DEGs between BM AML cells from control and CAP diet mice. (J) KEGG and GO pathway analysis of upregulated DEGs in the CAP diet versus the control. (K) Frequency of LSCs in BM 18 days post-transplantation (*n* = 6–7). (L) Frequency of early apoptosis AML cells in BM 18 days post-transplantation (*n* = 4). (M) Representative flow cytometry plots showing cell cycle analysis using Ki-67 and Hoechst 33342 staining. (N) Cell cycle distribution of BM AML cells from control and CAP diet mice (*n* = 4). Statistical analysis: (B, C, F, H, K, L, N) unpaired t-test; (D) paired t-test; (E) survival curve comparison. Data are presented as mean ± SD. Significance: ns, not significant, *P < 0.05, **P < 0.01, ***P < 0.001, ****P < 0.0001.

To assess the impact of nociceptive nerve activation, we analyzed transcriptomes of AML cells from mice fed a dietary capsaicin. Compared to controls, the CAP diet group showed upregulation of 835 genes and downregulation of 518 genes (Fig. 5I). Upregulated pathways were predominantly associated with immunoglobulin production and production of immune molecular mediators (Fig. 5J), indicating enhanced immune response. Concurrently, pathways related to myeloid differentiation—including mononuclear, leukocyte, myeloid, and monocyte differentiation—were elevated (Fig. 5J), indicating the role of capsaicin diet in promoting differentiation of LSCs. Lower frequency of LSCs in BM further validated the reduced LSCs burden after nociceptive nerve activation by dietary capsaicin (Fig. 5K; Fig. S4C). These transcriptomic profiles suggest that activation of nociceptive nerves through dietary capsaicin exerts anti-leukemic effects by bolstering immune responses and promoting LSCs differentiation in AML cells.

### 3.9 Dietary capsaicin enhances apoptosis and induces cell cycle arrest in AML cells

Since transcriptomic analysis revealed altered apoptotic signaling pathway and mitotic cell cycle pathway following neural manipulation (Fig. 5J; Fig. S2D), we further evaluated the effect of dietary capsaicin on apoptosis and proliferation. Annexin V and DAPI staining assay showed the proportions of apoptotic AML cells and LSC in BM were significantly higher in the CAP diet group than in controls (Fig. 5L; Fig. S4D–H). Similarly, in the SP, significantly higher frequencies of apoptotic AML cells were observed in the CAP diet group (Fig. S4I–K). Cell cycle analysis using Ki-67 and Hoechst 33342 staining revealed that the CAP diet group exhibited a higher percentage of AML cells in G₀ phase and a lower proportion in G₁ phase (Fig. 5M–N; Fig. S4L). These results demonstrate that dietary capsaicin impairs the proliferative capacity of AML cells. In summary, dietary capsaicin reduces leukemic burden by enhancing apoptosis and inducing cell cycle arrest in AML cells within the BM and SP.

### 3.10 CGRP inhibits leukemia progression by suppressing the TNF-α pathway in AML cells

Previous studies have suggested that AML cells in metaphysis exhibit an up-regulated TNF signaling pathway compared to those in diaphysis [24], which aligns well with the distribution of nociceptive nerve and preferential niche of AML cells. Transcriptomic analysis in our study further showed that the TNF signaling pathway is the most prominently altered pathway in AML cells after denervation (Fig. 4I). Consistent with prior studies highlighting the critical role of TNF-α in leukemia progression and the inhibitory role of CGRP in TNF‐α production and NF‐κB activation [[25], [26], [27]], we observed that nociceptive denervation significantly increased TNF-α levels in BM, whereas dietary capsaicin markedly reduced TNF-α (Fig. 6A). Although TNF-α is primarily derived from monocytes and macrophages, evidence also indicates that autocrine TNF-α can promote AML cell proliferation and progression [27]. Accordingly, we detected elevated TNF-α expression in AML cells from denervated mice, whereas TNF-α levels were lower in AML cells from mice with activated nociceptive nerves (Fig. 6B). Further analysis of the downstream NF‑κB pathway showed increased phosphorylation of NF‑κB in AML cells from denervated mice and reduced phosphorylation in cells from mice with activated nerves (Fig. 6C). These findings suggest that nociceptive nerves restrict leukemia progression by suppressing autocrine TNF-α in leukemia cells, thereby limiting activation of the NF‑κB pathway.

**Fig. 6:**
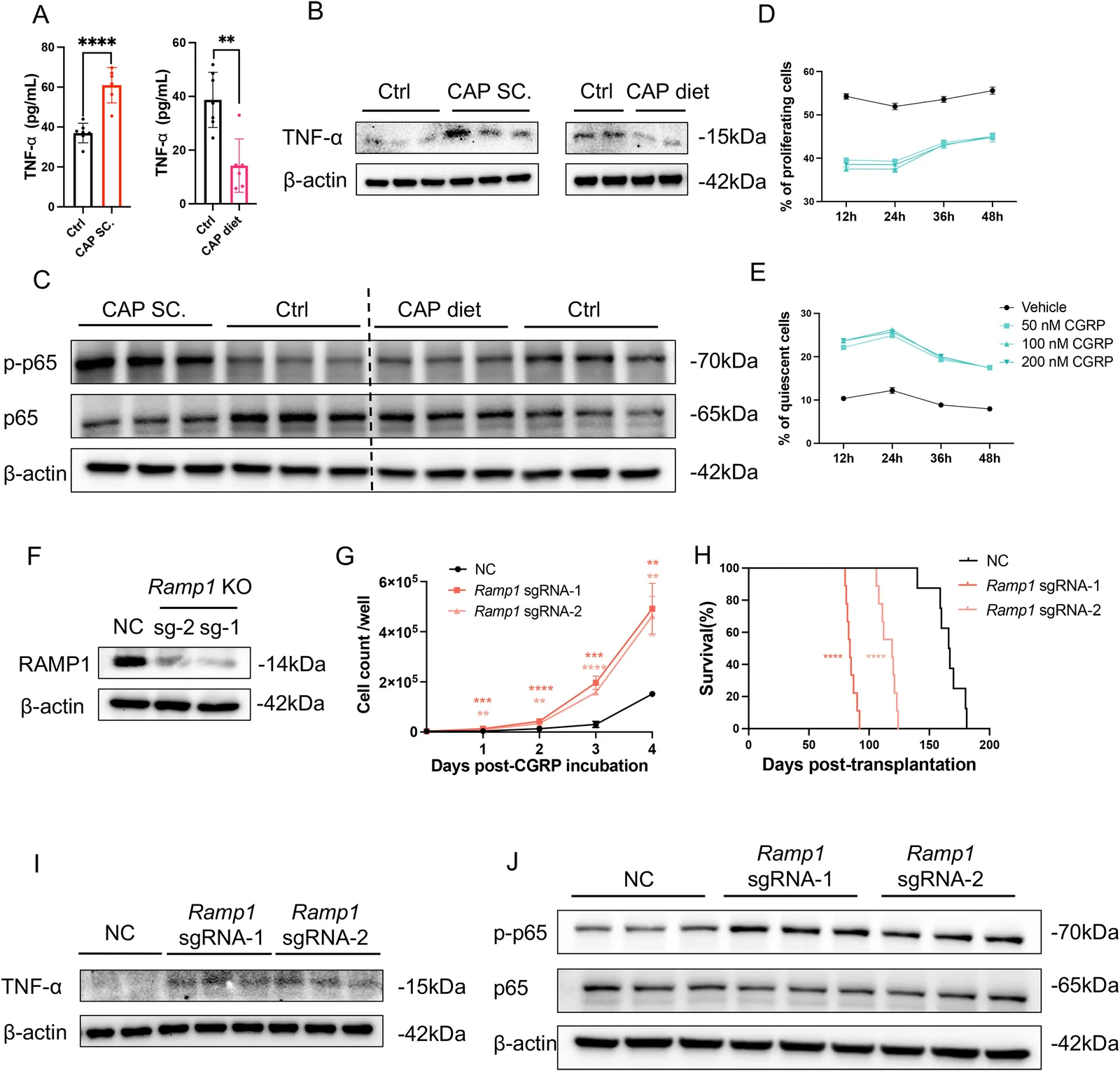
**CGRP suppresses the TNF signaling pathway in AML cells.** (A) Concentration of TNF-α in BMEF (*n* = 6–7). (B) Expression of TNF-α in AML cells after neural manipulation. (C) Phosphorylation of NF‑κB in AML cells after neural manipulation. (D) Frequency of proliferating AML cells after CGRP administration (*n* = 4). (E) Frequency of quiescent AML cells after CGRP administration (*n* = 4). (F) Expression of RAMP1 in AML cells after RAMP1-KO by CRISPR-Cas9. (G) Growth curves of NC and RAMP1-KO AML cells post-CGRP incubation (*n* = 3). (H) Overall survival of NC and RAMP1-KO AML models (*n* = 8–9). (I) Expression of TNF-α in NC and RAMP1-KO AML cells. (J) Phosphorylation of NF-κB in NC and RAMP1-KO AML cells. Statistical analysis: (A, G) unpaired t-test; (H) survival curve comparison. Data are presented as mean ± SD. Significance: ns, not significant, *P < 0.05, **P < 0.01, ***P < 0.001, ****P < 0.0001.

To directly assess the regulatory role of nociceptive nerves on AML cells, we incubated Fucci‑labeled AML cells with the neuropeptide CGRP and observed that CGRP directly altered their cell cycle distribution (Fig. 6D). To further validate the function of CGRP, we knocked out RAMP1—a key component of the CGRP receptor complex—in leukemia cells by CRISPR‑Cas9 (Fig. 6F). In vitro, RAMP1‑KO cells escaped CGRP‑mediated growth suppression and exhibited enhanced proliferation (Fig. 6G). Correspondingly, RAMP1‑KO AML mice showed significantly reduced overall survival (Fig. 6H). Mechanistically, RAMP1 deletion abrogated CGRP‑mediated inhibition of autocrine TNF-α, leading to sustained activation of NF‑κB and accelerated leukemia progression (Fig. 6I–J). In summary, our results demonstrate that nociceptive nerves release CGRP to suppress autocrine TNF-α in leukemia cells, thereby restraining NF‑κB activation and limiting leukemia progression.

## 4 Discussion

Our study demonstrates that the nociceptive nervous system, a key component of the BM and splenic microenvironment, plays a previously underappreciated yet critical role in suppressing AML progression. We provide anatomical and functional evidence that MLL-AF9-induced AML actively remodels the neuro-microenvironment at an early disease stage, resulting in substantial loss of both sympathetic and nociceptive nerves. Spatial mapping of neural architecture revealed regional specialization in bone marrow innervation, with the distal metaphysis serving as a neural-sparse niche that supports heightened leukemic proliferation. The preferential accumulation and expansion of AML cells in these neural-deficient regions suggest that neural-derived signals may normally inhibit leukemic growth, and that nerve loss fosters a permissive niche for disease progression. This observation aligns with emerging concepts of niche engineering in leukemia, whereby malignant cells actively remodel their microenvironment to support expansion [13].

Our results extend recent findings implicating the neuro-microenvironment in hematopoietic regulation [9,10] and malignancy [8]. Previous studies have focused predominantly on sympathetic nerves in hematological malignancies [8], and our study unveils a compelling organ-specific divergence in neural regulation of AML progression. While sympathetic denervation primarily exacerbated leukemic infiltration in the spleen without significantly affecting the BM, nociceptive denervation accelerated disease progression in both compartments. This distinction may reflect intrinsic differences in neural architecture and signaling milieu between these organs. The spleen is predominantly innervated by sympathetic fibers and harbors fewer nociceptive nerves, which likely renders splenic leukemia more sensitive to sympathetic modulation. In contrast, the bone marrow maintains a more balanced innervation by both nerve types, allowing nociceptive signaling to exert broader regulatory control over leukemic growth and hematopoietic homeostasis. These findings underscore the importance of considering organ-specific neuro‑microenvironments when designing neural‑targeted therapeutic strategies for AML.

A key finding of this study is the role of nociceptive nerves in AML progression. Pharmacological ablation of nociceptive nerve accelerated leukemic progression, increasing LSC burden nearly 5-fold in BM and 11-fold in SP. Conversely, activation of nociceptive nerves via dietary capsaicin significantly delayed leukemia onset, reduced leukemia burden, and improved survival. Additionally, this study provides new insights into the role of nociceptive nerves in regulating stressed hematopoiesis under AML conditions. Nociceptive denervation led to significant retention of HSCs in BM despite enhanced AML infiltration, while the spleen—though enlarged—contained fewer HSPCs. These results indicate that nociceptive denervation impairs HSPC mobilization and extramedullary hematopoiesis, thereby disrupting a crucial compensatory mechanism for bone marrow failure and contributing to reduced overall survival. Thus, restoring nociceptive nerve function may therefore be essential to curbing AML progression and sustaining compensatory hematopoiesis at extramedullary sites.

The protective effect of nociceptive nerve activation through dietary capsaicin suggests novel therapeutic approaches that could complement conventional chemotherapy by targeting the neural-leukemic niche. Several key questions, however, remain unanswered. Previous studies have shown that drug-resistant and relapsed leukemic stem cells often overexpress CALCRL [16]. However, CALCRL has multiple ligands beyond CGRP, and some evidence suggests that adrenomedullin—not CGRP—may be the ligand associated with CALCRL-mediated drug resistance and poor prognosis in AML [17]. Besides, nociceptive nerves release multiple neurotransmitters—including CGRP, substance P, and non-peptidergic transmitters—that form a complex signaling network engaging various immune and niche cells through multiple immune-regulatory pathways [[28], [29], [30], [31], [32]]. While our transcriptomic and mechanistic studies demonstrate that neural interventions remodel immune pathways and that CGRP regulates TNF signaling in AML cells, we believe that neural regulation of AML progression extends beyond these direct CGRP effects. The broader influence of neural signaling, encompassing both direct effects on leukemic cells and indirect modulation of the microenvironment, warrants further elucidation.

## 5 Conclusion

Our findings suggest that nociceptive nerves serve as important regulators of AML development and highlight neuro-modulatory interventions as promising therapeutic avenues. The ability of dietary capsaicin to delay AML progression via nociceptive nerve activation suggests that dietary capsaicin may represent a potentially complementary approach to conventional AML therapies. Future studies investigating combined neural activation and chemotherapy may lead to innovative treatment approaches that simultaneously target leukemic cells and the neural niche.

## Data availability

The raw RNA-seq data generated in this study have been deposited in the NCBI Sequence Read Archive (SRA) under the BioProject accession number PRJNA1312165. Processed gene expression matrices and analysis-ready data are available from the corresponding author upon reasonable request.

## CRediT authorship contribution statement

**Honglin Duan:** Writing – review & editing, Writing – original draft, Visualization, Validation, Supervision, Software, Resources, Project administration, Methodology, Investigation, Formal analysis, Data curation, Conceptualization. **Pujiao Li:** Investigation. **Linmin Zhang:** Investigation. **Yanling Lv:** Software. **Meng Yang:** Investigation. **Yicheng He:** Investigation. **Shangda Yang:** Funding acquisition, Methodology. **Shihong Lu:** Funding acquisition. **Tao Cheng:** Writing – review & editing, Supervision, Investigation, Funding acquisition, Conceptualization. **Hui Cheng:** Writing – review & editing, Supervision, Investigation, Funding acquisition, Conceptualization.

## Declaration of competing interest

The authors declare that they have no conflicts of interest in this work.

## Appendix Supplementary materials

Image, application 1

Image, application 2
