# Supplementary material for: Modulation of nociceptive nerve activity as a novel therapeutic strategy in acute myeloid leukemia
Source: Fundam Res. 2026 Mar 28;6(5):3507–18. doi: 10.1016/j.fmre.2026.03.012 (PMC13644478; doi:10.1016/j.fmre.2026.03.012)

Figure S1. Sympathetic denervation alters the infiltration of AML cells in spleen instead of BM.

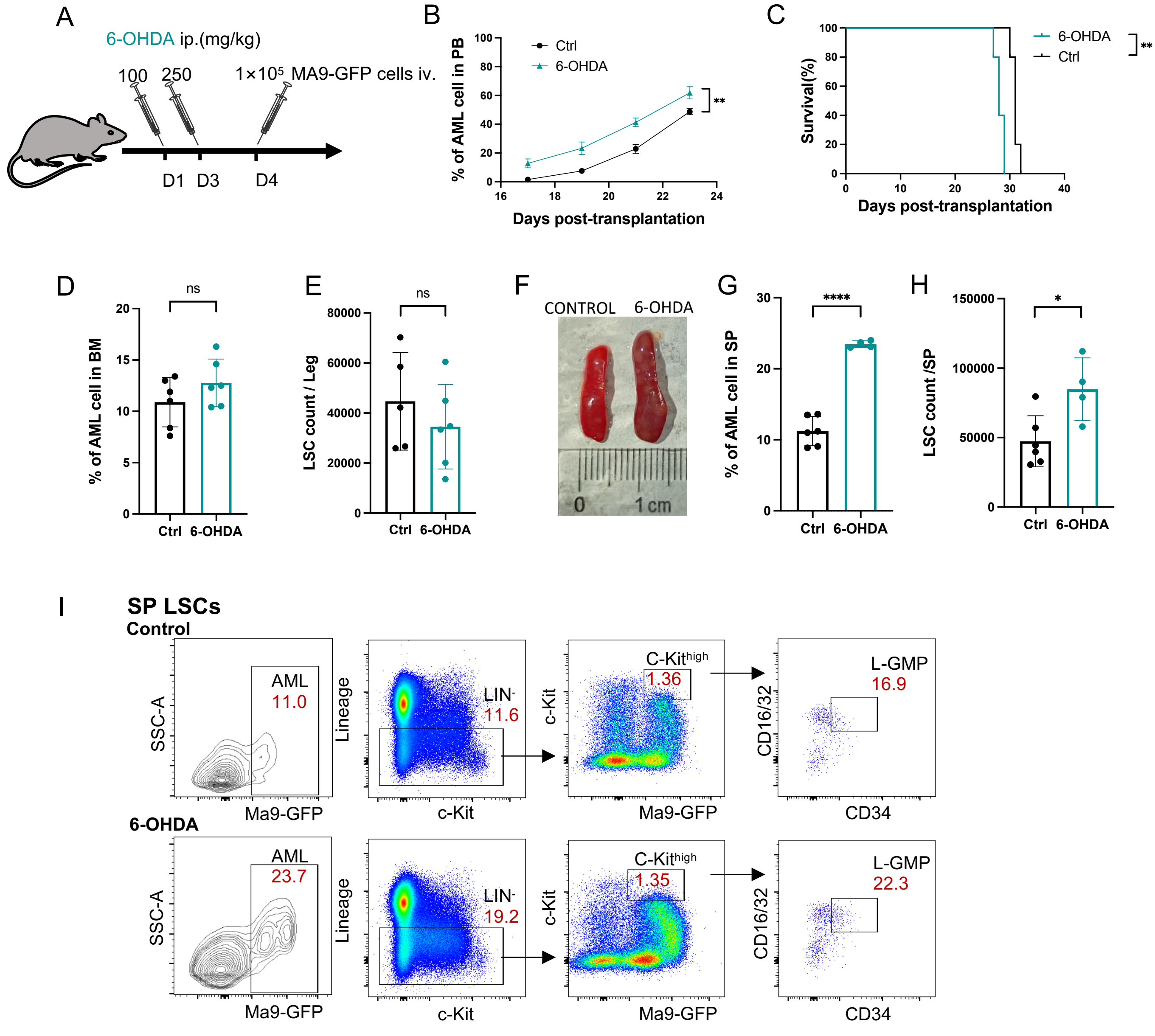

Figure S2. Nociceptive denervation facilitates invasion of AML cells in BM and SP.

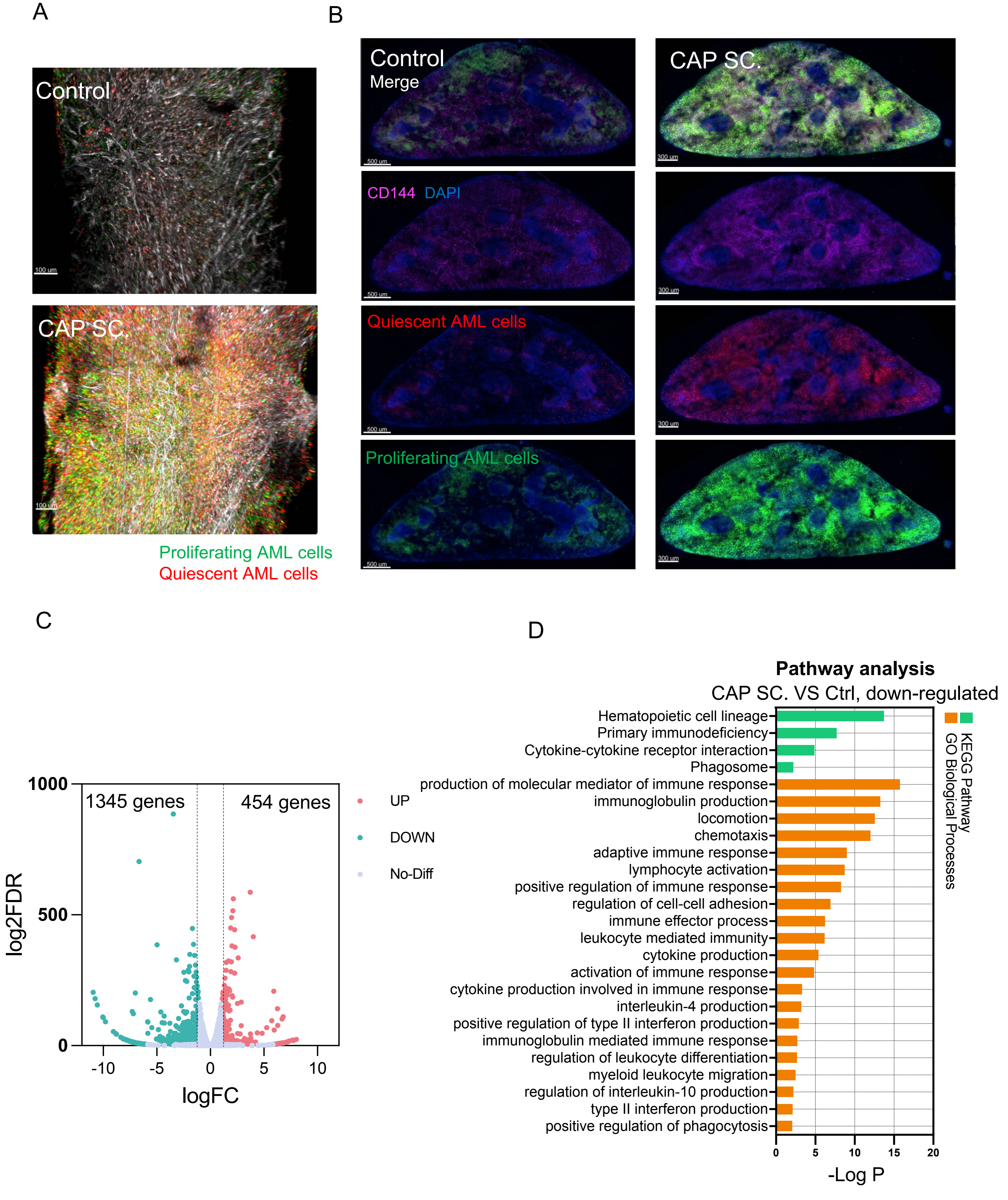

Figure S3. Nociceptive denervation disturbs stressed hematopoiesis.

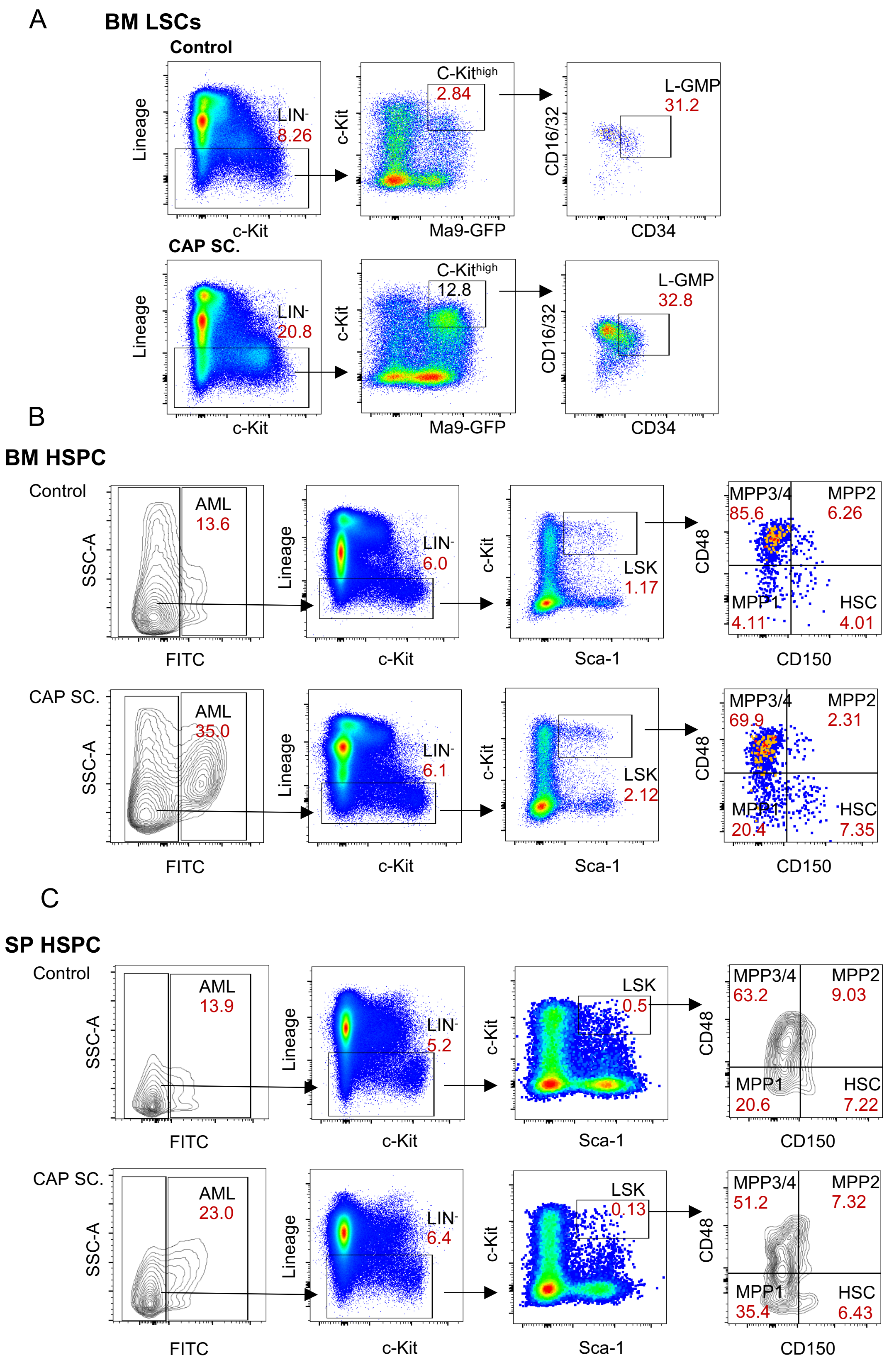

Figure S4. Effects of spicy food diet on apoptosis and proliferation of AML cells.

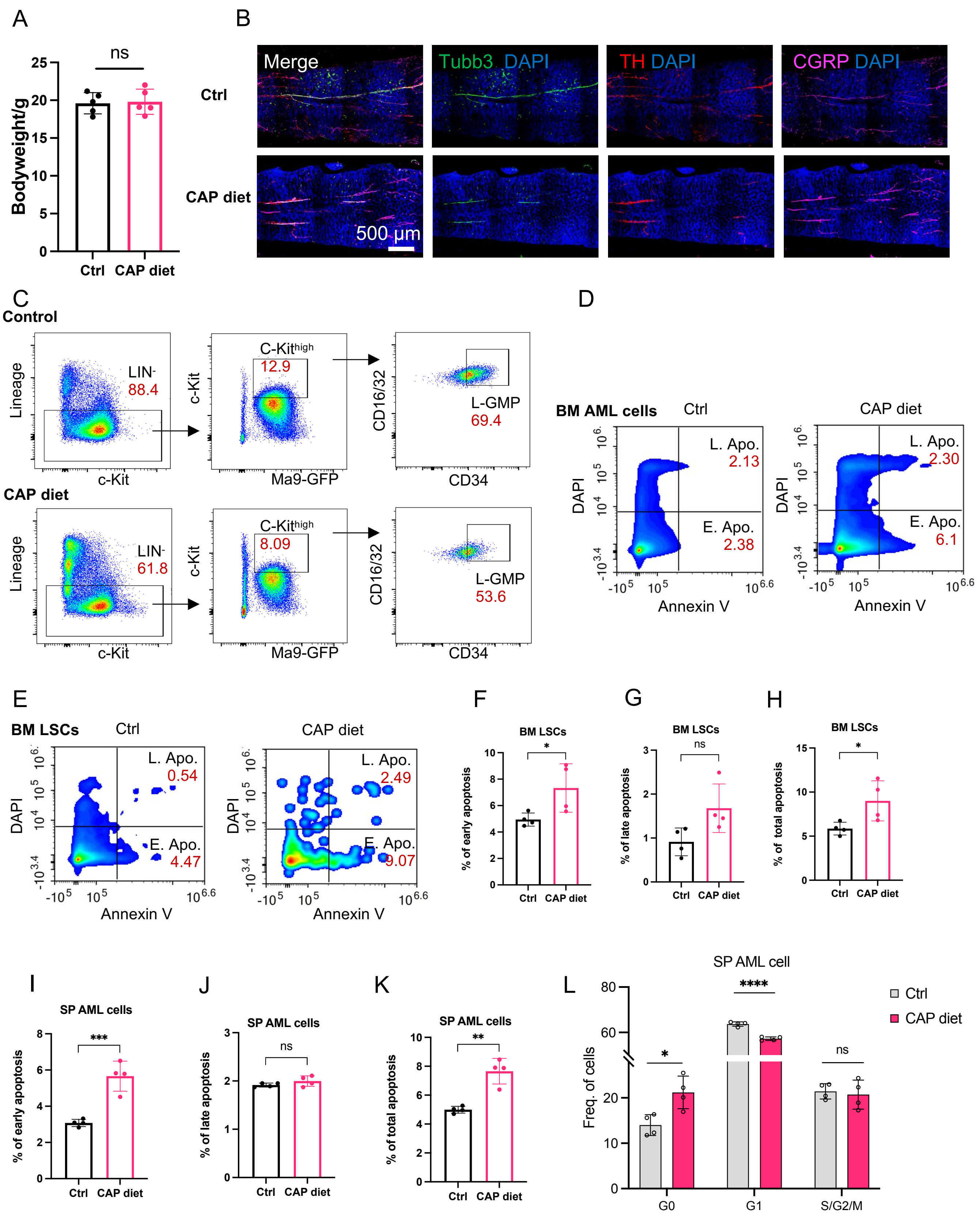

Supplement: Supplementary file 1 [file mmc1.pdf]
