# Supplementary material for: Modulation of nociceptive nerve activity as a novel therapeutic strategy in acute myeloid leukemia
Source: Fundam Res. 2026 Mar 28;6(5):3507–18. doi: 10.1016/j.fmre.2026.03.012 (PMC13644478; doi:10.1016/j.fmre.2026.03.012)
Supplement: Supplementary file 2 [file mmc2.docx]

**Supplementary Figure Legends**

**Figure S1. Sympathetic denervation alters the infiltration of AML cells in spleen instead of BM.**

(A) Schematic of the denervation of sympathetic nerves by 6-OHDA.

(B) Denervation of sympathetic nerves shows higher frequency of AML cell in PB (n=4-6).

(C) Overall survival of control and 6-OHDA treated AML models (n=5).

(D) Frequency of AML cells in BM 17 days post-transplantation (n=6).

(E) LSC count per leg 17 days post-transplantation (n=6).

(F) Representatives images of AML spleen 17 days post-transplantation.

(G) Frequency of AML cells in spleen 17 days post-transplantation (n=4-6).

(H) LSC count in spleen 17 days post-transplantation (n=4-6).

(I) Representative flow cytometry plots showing gating strategy of LSCs (L-GMPs) in SP.

Statistical analysis: (B) paired t test; (C) survival curve comparison. (D, E, G, H) unpaired t test. Data are presented as mean ± SD. Significance: ns= not significant, **P* < 0.05, ***P* < 0.01, ****P* < 0.001, *****P* < 0.0001.

**Figure S2. Nociceptive denervation facilitates invasion of AML cells in BM and SP.**

(A-B) Representative confocal images showing the proliferating (green) and quiescent (red) AML cells in BM (A) and SP (B) at early stage of AML.

(C) Volcano plots showing DEGs between BM AML cells from control and CAP SC. mice.

(D) KEGG and GO pathway analysis of downregulated DEGs in CAP SC. versus control.

**Figure S3. Nociceptive denervation disturbs stressed hematopoiesis.**

(A) Representative flow cytometry plots showing LSCs (L-GMPs) in BM from control and CAP SC. mice.

(B-C) Representative flow cytometry plots showing gating strategy of HSPCs in BM (B) and SP (C) from control and CAP SC. mice 17 days post-transplantation.

**Figure S4. Effects of spicy food diet on apoptosis and proliferation of AML cells.**

(A) Body weight of control and CAP diet mice (n=5).
(B) Representative confocal images showing Tubb3^+^, TH^+^ and CGRP^+^ nerve fiber in CAP diet group and control group.

(C) Representative flow cytometry plots showing LSCs (L-GMPs) in BM from control and CAP diet mice.

(D-E) Representative flow cytometry plots showing apoptosis of BM AML cells (D) and LSCs (E) from control and CAP diet mice.

(F-H) Frequency of early (F), late (G), and total (H) apoptotic LSCs in BM from control and CAP diet mice (n=4).

(I-K) Frequency of early (I), late (J), and total (K) apoptotic AML cells in SP from control and CAP diet mice (n=4).

(L) Cell cycle distribution of SP AML cells (n=4).

Statistical analysis: unpaired t test. Data are presented as mean ± SD. Significance: ns= not significant, **P* < 0.05, ***P* < 0.01, ****P* < 0.001, *****P* < 0.0001.
